# Reducing weight gain in people with schizophrenia, schizoaffective disorder, and first episode psychosis: describing the process of developing the STructured lifestyle Education for People With SchizophrEnia (STEPWISE) intervention

**DOI:** 10.1186/s40814-018-0378-1

**Published:** 2018-12-17

**Authors:** Marian E. Carey, Janette Barnett, Yvonne Doherty, Katherine Barnard, Heather Daly, Paul French, Rebecca Gossage-Worrall, Michelle Hadjiconstantinou, Daniel Hind, Jonathan Mitchell, Alison Northern, John Pendlebury, Shanaya Rathod, David Shiers, Cheryl Taylor, Richard I. G. Holt, On behalf of the STEPWISE Research Group, Richard I. G. Holt, Katharine Barnard, Rebecca Gossage-Worrall, Mike Bradburn, Daniel Hind, David Saxon, Lizzie Swaby, Paul French, David Shiers, John Pendlebury, Stephen Wright, Glenn Waller, Paul McCrone, Tiyi Morris, Charlotte Edwardson, Kamlesh Khunti, Melanie Davies, Marian Carey, Yvonne Doherty, Alison Northern, Janette Barnett, Richard Laugharne, Christopher Dickens, Kathryn Greenwood, Sridevi Kalidindi, Fiona Gaughran, Shanaya Rathod, Najma Siddiqi, Angela Etherington, David Shiers

**Affiliations:** 1 Leicester Diabetes Centre, University Hospitals of Leicester NHS Trust, Leicester, UK; 2 Department of Health Sciences, University of Leicester, Leicester, UK; 3 Faculty of Health & Social Science, Bournemouth University, Bournemouth, UK; 4 Psychosis Research Unit, Greater Manchester Mental Health NHS Foundation Trust, Manchester, UK; 5 Clinical Trials Research Unit, University of Sheffield, Sheffield, UK; 6 Sheffield Health and Social Care NHS Foundation Trust, Sheffield, UK; 7 Southern Health NHS Foundation Trust, Southampton, UK; 8 Human Development and Health Academic Unit, University of Southampton, Southhampton, UK; 9 Kairos Communications and Research Ltd, 69 Avenue Road Extension, Leicester, LE2 3EP UK

**Keywords:** Schizophrenia, Psychosis, Antipsychotic, Obesity, Lifestyle, Self-management, Structured education, Weight management

## Abstract

**Background:**

Obesity is twice as common in people with schizophrenia as the general population and associated with significantly worsened psychiatric and physical health. Despite National Institute for Health and Care Excellence guidelines for the management of psychosis recommending that mental health services offer lifestyle programmes to people with schizophrenia to improve physical health, this is not currently occurring. The aim of the STEPWISE research programme was to develop a lifestyle intervention addressing obesity and preventing weight gain in people with schizophrenia, schizoaffective disorder, or first episode psychosis taking antipsychotic medication, through an approach and fundamental principles drawn from existing diabetes and diabetes prevention interventions. This paper describes the often under-reported process of developing such an intervention from first principles.

**Methods:**

Following an extensive literature review, an iterative cycle of development with input from people with schizophrenia, mental healthcare professionals, facilitators, and other stakeholders, a new weight management intervention for the target group was developed. A set of four core weekly sessions was piloted in Sheffield, followed at 3-monthly intervals by three booster sessions and telephone support contact once every 2 weeks, to form an intervention lasting 12 months. Facilitators were provided with a 4-day training package to support delivery of the intervention.

**Results:**

This paper reports the process of development, including challenges and how these were addressed. It describes how user input influenced the structure, topics, and approach of the intervention. The outcome of this process was a feasible and acceptable lifestyle intervention to support people with schizophrenia, schizoaffective disorder, or first episode psychosis to manage their weight. This pilot provided opportunities for refinement of the intervention and facilitator training prior to testing in a multi-centre randomised controlled trial. Key findings from the pilot were linked to accessibility, focus, uptake, and retention, which influenced session length, travel arrangements, refreshment, breaks, and supporting tools to incentivise participants.

**Conclusions:**

The STEPWISE intervention has been evaluated in a randomised controlled trial in 10 mental health trusts in England, and the results will be published in the British Journal of Psychiatry and the NIHR Journals Library.

**Trial registration:**

ISRCTN19447796. Date registered: 20/03/2014

## Background

Schizophrenia is a major psychiatric disorder that affects approximately 1% of the population and can severely alter individuals’ perceptions, behaviour and cognition [1]. On average people with schizophrenia die 10–20 years prematurely, with cardiovascular disease being the most common cause of death [2], and obesity and overweight being significant contributors to this morbidity and mortality. Obesity occurs early in the natural history of schizophrenia; a significant proportion of people with first episode psychosis are overweight prior to any treatment and weight gain can accelerate within weeks of treatment initiation [3]. This trajectory continues and around two-thirds of individuals experience clinically significant weight gain (> 7%) over the first 12 months of treatment [3]. This rapid weight gain is associated not only with adverse lifestyle factors, such as poor diet and high sedentary behaviour, but is also linked to the psychiatric medication [4]. Weight gain is a common side effect of antipsychotics, affecting between 15 and 72% of patients [5]. Propensity to cause weight gain differs between antipsychotics but no agent should be considered as weight-neutral [6].

There is an increasing awareness of the challenge of obesity in people with schizophrenia and recent NICE guidelines have recommended that combined healthy eating and physical activity programmes to address overweight and obesity are available to people with psychotic illness [7]. Despite the clear clinical need to address obesity, there remains a significant gap in how mental health services currently support people with schizophrenia with their weight management [8].

The aim of the STEPWISE project, and the focus of the Intervention Development Study (IDS) group, was to develop a sustainable evidence-based programme to support people with schizophrenia, schizoaffective disorder or first episode psychosis with their weight management, in such a way that it was acceptable, feasible to deliver, deliverable within available resources, and effective.

The resulting STEPWISE (STructured lifestyle Education for People With SchizophrEnia) intervention was developed by a team from the Leicester Diabetes Centre, in conjunction with expert colleagues and with patient and public involvement and engagement. Following a pilot phase, the intervention was tested in a multi-centre randomised controlled trial in England and the results will be published in the British Journal of Psychiatry and NIHR Journals Library.

## Methods

The intervention development took place as the first phase of an NIHR Health Technology Assessment project (12/28/05). Ethics approval was granted by South Yorkshire Research Ethics Committee (14/YH/0019).

### Literature review

A meta-analysis of non-pharmacological interventions published in 2012 by Caemmerer and colleagues, conducted across 17 studies and published prior to the commencement of the intervention development, reported the benefit of behavioural interventions in significantly reducing weight gain and body mass index (BMI) in people receiving antipsychotic medication, compared to controls [9]. The development team revisited this systematic review by rerunning the search strategy across the PsycInfo, MEDLINE, PubMed, CINAHL, and Cochrane Library databases, using the original search terms, ‘weight’, ‘antipsychotic’, and ‘intervention’ plus ‘behavioural’, ‘psychoeducation,’ exercise’, or ‘cognitive’. The Caemmerer meta-analysis was particularly pertinent as it included RCTs of non-pharmacological interventions focused on preventing or reducing antipsychotic associated weight gain. The purpose of re-running the search strategy was to ensure no additional and relevant interventions had been published in the interim, to determine the key features of weight management programmes identified in the review, and to clarify common elements that could be incorporated into the STEPWISE programme.

### Development of a theoretical framework

Concurrent with the literature review and informed by it, a theoretical framework was developed using currently recommended processes [10, 11]. The following three key areas were considered core to weight management interventions in the target group:

- ▪ Behaviour change theory;
- ▪ Psychological processes core to weight management;
- ▪ Living with psychosis.

### Intervention development

The STEPWISE programme was initially planned as an adaptation of an existing diabetes prevention programme, ‘Let’s Prevent Diabetes’, which is a lifestyle change programme for people at increased risk of type 2 diabetes that demonstrated modest but significant benefits in biomedical and lifestyle outcomes [12].

**Fig. 1:**
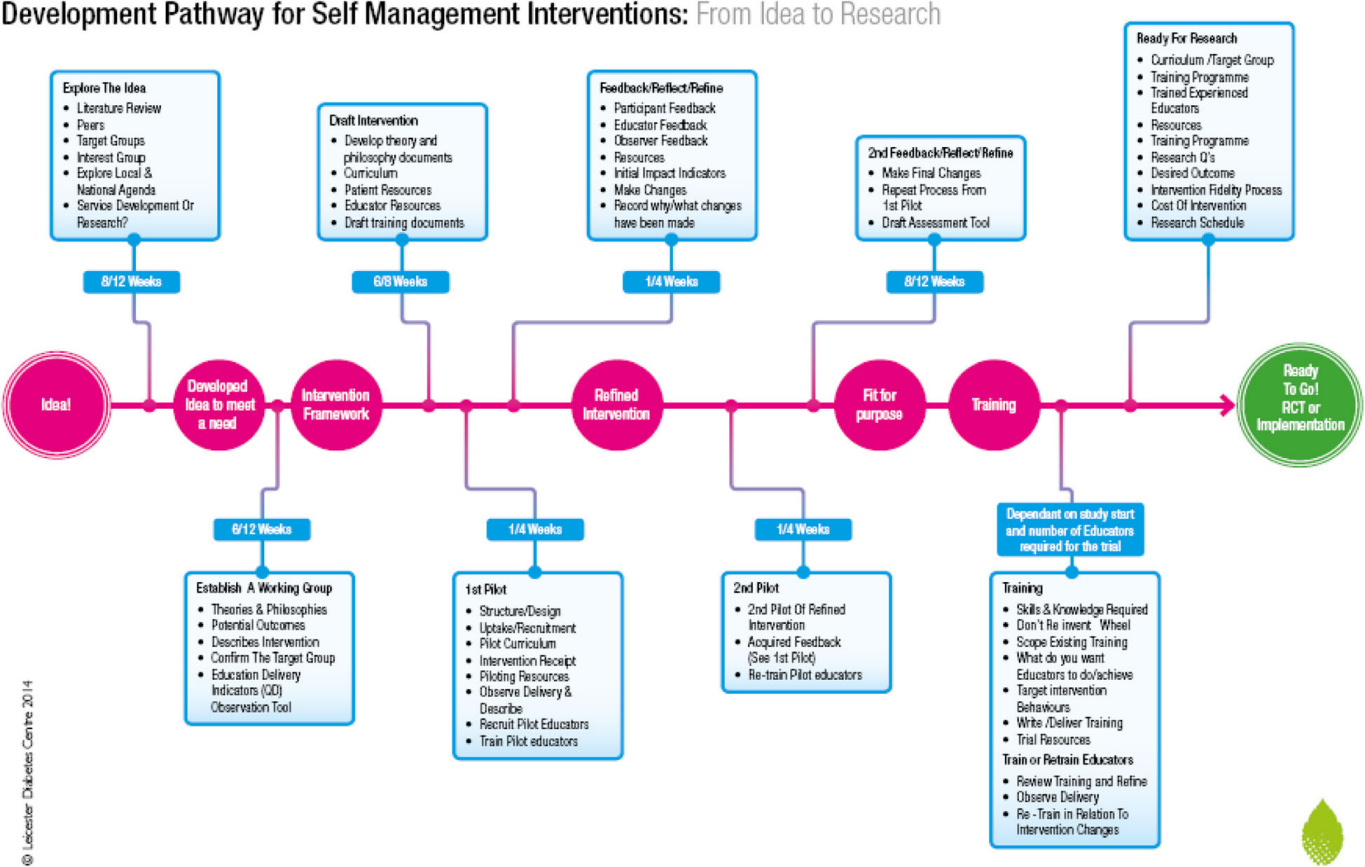
Leicester development pathway for self-management interventions

It soon became apparent from initial meetings with stakeholders, the literature review, and expert opinions of clinicians and practitioners actively providing local weight management interventions for people with psychosis, that although the guiding principles underpinning development of the Let’s Prevent programme remained relevant, the programme itself was not suitable for STEPWISE target participants. The Let’s Prevent programme focuses upon prevention of diabetes messages, rather than weight loss. It was delivered in a single 6-h session which was considered too lengthy for people with schizophrenia who often experience concentration difficulties. Furthermore, despite incorporating an annual refresher session, and 3-monthly telephone support contacts, Let’s Prevent did not provide the length of contact time which evidence suggested was necessary to support weight loss/behaviour change in this client group [9]. The IDS group made the decision to return to the proven guiding principles for developing self-management interventions, which have a philosophical and theoretical basis, in order to develop the STEPWISE intervention [10, 11], using a pathway established in Leicester and based on a formal framework [13] (Fig. 1). Using this framework, a multi-disciplinary team comprising a consultant clinical psychologist, two specialist educationalists, a dietitian, and creative designer, with additional input from the IDS group, developed a prototype STEPWISE intervention. This sought to promote autonomous problem solving around food and physical activity choices, with a specific focus on relapse prevention and weight improvement.

The intervention development incorporated three stages: (1) prototype development; (2) pilot of the prototype including incorporation of amendments and adaptation; and (3) training of facilitators. This paper describes the first two stages.

#### Prototype development

Central to the prototype development was the collaboration between a team with expertise in the development of obesity and lifestyle intervention programmes, mental healthcare professionals, researchers with specialist knowledge of the needs of people with schizophrenia and psychosis**,** and the input of service users and participants throughout the pilot.

Following the literature review, the team sought the input of service users in both Sheffield and Leicester, but due to organisational circumstances outside of the team’s control, it was not possible to identify people with schizophrenia willing to meet with the developers at this time. Service user input was therefore initially drawn from the various published studies in the Caemmerer review [9] in which this was reported. Participants of the pilot sessions and service users not participating in the pilot provided ongoing feedback and input during the pilot stage, as is described later in this paper.

The programme developers were able to meet with an expert practitioner with experience of delivering a weight management group for people with schizophrenia in Salford. The developers also sought the opinions of health care professionals working in mental health drawn from Sheffield and from within the investigator team. Together with the findings of the literature review, these contributions informed the design of the prototype intervention, its content, delivery, and logistics.

The ‘shape’ of the intervention, choice, and ordering of topics within sessions and from one session to another was created by following the Leicester Pathway (Fig. 1). A pathway of this kind is essential to developing programmes systematically, to ensure an iterative process that incorporates feedback as an ongoing component.

**Fig. 2:**
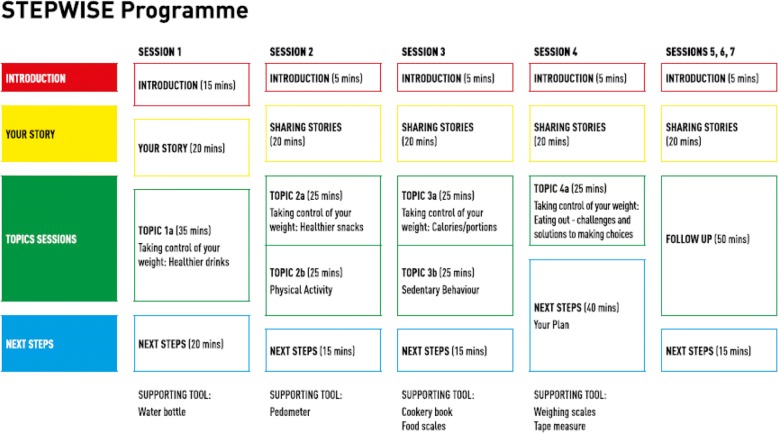
Outline of STEPWISE core sessions

The prototype intervention comprised four core group education sessions, to be delivered to small groups of 6–8 participants over four consecutive weeks, with a duration of 90 min per session. A breakdown of each session can be found in Fig. 2.

The intervention development team delivered the prototype sessions in Sheffield and were observed by four healthcare professionals identified as potential facilitators for that research site, to enable both developers and prospective facilitators to have insight into the workings of the programme. Facilitator feedback was essential in both confirming the appropriateness of content and resources as well as supporting the effectiveness and suitability of the delivery style and content of the programme. The delivery style, in particular, being facilitative and non-didactic, was an innovation to the mental health professionals involved in the study. The sessions were designed for delivery by trained facilitators from each of the ten mental health trust research ‘sites’ committed to participate in the subsequent randomised controlled trial. It had been planned that two facilitators in each site would deliver the intervention together during the trial, with at least one of the pair being a registered mental health professional and the other an individual with a professional background as either a registered mental health professional, mental health support worker, or healthcare assistant. We expected that both facilitators would have current experience of working with people with mental health issues and that at least one of them would have knowledge of antipsychotic medication.

The literature review indicated the importance of follow-up sessions and ongoing support. Consequently, once the core sessions had been established, a series of follow-up ‘booster’ sessions were added to take place at 4, 7, and 10 months after the start of the intervention (110 min per session) and subsequent to the core sessions. The booster sessions were extended by 10 min to allow more time for participants sharing stories, as the time between sessions was much greater than the core sessions. Participants would be additionally supported by telephone calls from the facilitator team once every 2 weeks. It was recognised that training to deliver any of these intervention elements would be essential for facilitators.

#### Pilot

**Fig. 3:**
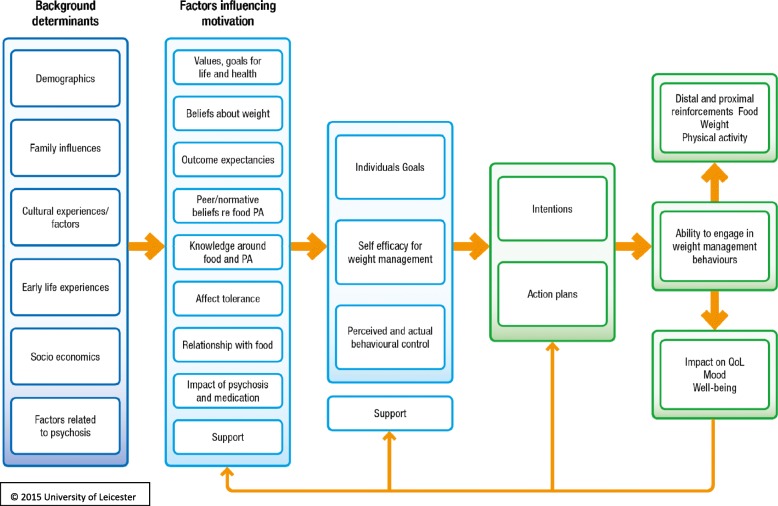
Theoretical framework of the STEPWISE intervention

The pilot testing of the intervention was undertaken in a community setting and comprised a cycle of four cohorts all taking place between May and December 2014 (Fig. 3). Biomedical and lifestyle outcomes were not collected during the pilot, since its purpose was to test the acceptability and feasibility of content and delivery and to collect feedback from participants to directly inform any necessary amendments and revisions to the intervention prior to the randomised controlled trial.

It was anticipated that the pilot would inform the training programme for facilitators and improve understanding of the obstacles and enablers to delivering the intervention in a real-world situation, as highlighted by Bartholomew and colleagues [11] and in accordance with the MRC framework for evaluating complex interventions [14].

The participants in the pilot sessions were recruited from community mental health teams from within Sheffield Health and Social Care NHS Foundation Trust. Using criteria agreed for the randomised controlled trial (Table 1), people were eligible to participate if they had a diagnosis of schizophrenia or schizoaffective disorder or first episode psychosis, were aged ≥ 18 years, and were receiving care through the Community Mental Health services in Sheffield. This was to ensure the intervention developed in line with the needs of potential trial participants. Participants were identified during routine clinic appointments and case note review, and given brief information about the study by the referring healthcare professional.

**Table 1:** Inclusion and exclusion criteria for STEPWISE feasibility pilot

Inclusion criteria	
1) Age ≥ 18 years old. There is no upper age limit. 2) Clinical diagnosis of schizophrenia or schizoaffective disorder (defined by ICD-10 codes F20 and F25) or first episode psychosis using case note review. There is no limit on the duration of illness for those with schizophrenia or schizoaffective disorder, but first episode psychosis is defined as less than 3 years since presentation to the mental health team 3) Patients being treated with an antipsychotic. For those with established schizophrenia or schizoaffective disorder, the treatment duration should be at least 1 month prior to entry in to the trial. 4) Ability to give written informed consent 5) Ability and willingness to attend and participate in a group education programme 6) Ability to speak and read English 7) Body mass index ≥ 25 kg/m^2^ or concerned about weight. For patients from South Asian and Chinese backgrounds, the BMI threshold is reduced to ≥ 23 kg/m^2^.	
Exclusion criteria	
1) Physical illnesses that could seriously reduce their life expectancy or ability to participate in the trial 2) A co-existing physical health problem that would, in the opinion of the PI, independently impact on metabolic measures. 3) Mental illnesses that could seriously reduce their ability to participant in the trial 4) Current pregnancy, plus mothers less than 6 months post-partum. 5) Conditions associated with significant weight gain, e.g., Cushing’s syndrome 6) Significant alcohol or substance misuse which, in the opinion of the PI, would limit the patient’s ability to participate in the trial. 7) A diagnosis or tentative diagnosis of psychotic depression or mania 8) A primary diagnosis of learning disability 9) Currently (or within past three months) engaged in a systematic weight management programme.	

Participants were recruited to one of the four pilot cohorts and invited to attend the four core sessions, each session lasting approximately 2 h, including a refreshment break. Sessions were delivered weekly, using a venue local to participants. Throughout the pilot phase, observation notes were taken at each session by an independent researcher, supplementing feedback from participants and facilitators, in order to review and evaluate the programme content, resources, and delivery, and to shape the final overall content and style of the intervention and its resources. Where possible and appropriate, changes were incorporated into the intervention during the pilot. For example, suggestions to improve logistics with taxi provision for participants introduced after cohort 2 sessions proved crucial to improving both uptake and retention in the subsequent pilot cohorts, recruitment in the subsequent RCT, and in contributing to training for facilitators.

##### Service user input and feedback

Although it was not possible to obtain user feedback during the prototype period, arrangements were more successful during the pilot. The last session of each of the four cohorts was planned in advance and notified to participants as an extended session allowing for 30–40 min of feedback.

Ethical approval was granted for the use of an audio recorder at the focus groups. However, at the first feedback session with participants, concerns, and apprehension regarding joining an audio recorded feedback session were raised, and a decision was made to forego audio recordings in all feedback sessions. Instead, information was documented in a systematic way by the researcher leading the feedback, an independent health psychologist. Where the group was larger, the support of an experienced and independent scribe was also employed.

Feedback from facilitators was audio recorded with consent and included both focus groups and 1:1 interviews, depending on availability and preference. Interviews with participants and facilitators took place separately.

An initial set of guided questions were used to prompt discussion with both sets of contributors, using two separate topic guides. Areas of discussion included experience travelling to and attending the sessions; the education programme itself; benefits of attending the programme; logistics of running programmes; training needs identified.

During the pilot phase, a user-led, Leicester-based local mental health support group comprising people with schizophrenia and other mental health conditions such as depression and bipolar disorder was identified as willing to provide a patient and public involvement and engagement perspective. Eleven people over the course of two group meetings, which were facilitated by two researchers closely involved in the prototype development, contributed to discussions around the suggested prototype curriculum, resources, and delivery logistics. The researchers also used these opportunities to discuss challenges and issues raised during the pilot, including problems of recruitment and retention.

Following completion of all 4 cycles of piloting, there was final refinement of the facilitator curricula and all participant materials and tools. A total of 60 community mental health professionals were subsequently trained in the 10 centres throughout England comprising the research sites in readiness for the randomised controlled trial.

## Results

### Literature review

At the outset of the development, the most recent systematic review and meta-analysis [9] confirmed the benefits of non-pharmacological interventions but was unable to identify differences across modalities, duration, and group versus individual delivery. The only difference identified was the benefit of outpatient over inpatient interventions. There was some indication that nutritional interventions may have a greater effect than cognitive behavioural therapy, but the authors concluded there was a such a great deal of overlap between interventions that distinctions were difficult to determine. Our literature search identified only one further paper [15] on aspects of weight gain associated with second generation antipsychotics. In general, the review found that interventions ran over a number of weeks ranging from 12 to 24 weekly sessions, in both groups and individually. There was a variation in the delivery of exercise with some providing specific cardio training; common dietary themes included reading food labels, switching drinks from full sugar to low calorie and healthy snacks. Other strategies incorporated eating more slowly and deliberately and recognising satiety. Several used ‘psychoeducation’ but further detail was not specified; a theoretical base was only reported in one study that specifically employed social cognition theory [16]. A more recent systematic review [17] on nutritional interventions concluded that early interventions and those led by dietitians showed a greater effect size. The description of each of the interventions in the systematic review were not of sufficient detail to allow these to be adapted or incorporated into the STEPWISE intervention.

### Theoretical framework

**Fig. 4:**
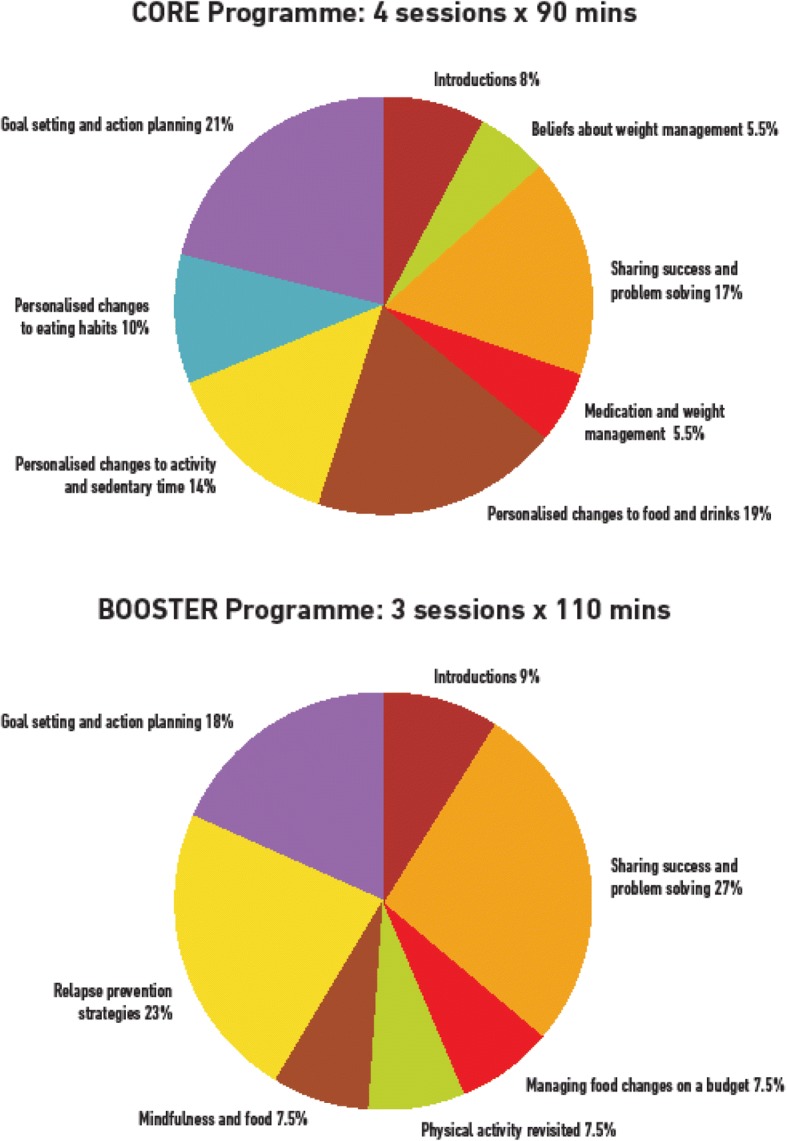
STEPWISE: time allocated to specific behavioural interventions

As the theoretical basis of the interventions was only specified in one paper despite this being widely considered to be good practice [11], the STEPWISE developers used the wider behaviour change and weight management literature to inform their approach. Factors specific to living with psychosis and taking antipsychotics were considered essential. These core factors determined the draft theoretical framework to guide the overall development of the intervention as shown in Fig. 3. This enabled the developers to ensure a focus on key hypothesised problem behaviours, be clear about the receipt of the intervention by the participants [18], and thus apply appropriate behaviour change techniques [19, 20]. The intervention was inspired by a number of theories (Fig. 3), but systematically employed three as demonstrated in Table 2 and was coded using the behaviour change taxonomy [20]. Figure 4 provides a visual representation of the percentage time allocated to each of the core behaviour change interventions.

**Table 2:** Development of the intervention (underpinning theories)

Identified target behaviour/problem	Theory	Participant receipt and potential behavioural outcome	Intervention on the STEPWISE course	Mapping to behavioural taxonomy (Michie et al. [19, 20])
Erroneous belief about weight problems.	Self-regulation theory (Leventhal, 1984) [28] Specifically illness representations around weight management • Signs of a weight problem • Causes • Consequences • Treatment • How long it will last	To have identified their own potential erroneous beliefs and questioned these in order to directly influence their decisions around weight management.	Your story session Elicit participant’s beliefs about what caused their weight problem, what ‘treatment’ would help to manage it, the consequences for them and their health. Topic sessions Information sessions throughout the course.	Not completely specified but included in • Information about health consequences • Framing/reframing
Low levels of confidence around being able to engage in successful weight management possibly related to multiple unsuccessful attempts at sustained weight loss.	Self-efficacy (Bandura 1977, 1997) [29, 30] • Mastery (previous successful attempts of the behaviour) • Modelling (observing others engaging in the behaviour) • Verbal persuasion (talking through the process of change expecting success) • Emotional arousal (managing the anxiety around change and fear of failure)	Increased belief in their ability to engage successfully in weight management	Sharing stories session Eliciting what has gone well in terms of behaviour change, problem solving around challenges, and observing others’ successes and problem solving. Discussing feelings as activators and barriers to change. Next STEPS Action planning, problem solving, setting small graded tasks	• Focus on past successes • Self-monitoring of behaviour outcomes of behaviour and consequences • Instruction on how to perform the behaviour • Graded tasks • Behavioural experiments • Credible source • Habit reversal • Review behavioural goals • Social comparison • Focus • Goal setting • Action planning • Problem solving • Information about antecedents • Information about emotional consequences • Reduce negative emotion • Self-incentive • Self-reward
Strong cues to previous behaviours and thus high likelihood of relapse	Relapse prevention model (Marlatt and Gordon 1985) [31] • High-risk situations with strong cues need to be managed by avoidance or coping strategies. • Coping strategies need to be prepared in advance • Management of relapse will result in increased self-efficacy	Reviewed the situations that would most likely result in relapse. Developed plans of how to manage these when they occur. View relapse as a natural part of the change process and as an opportunity to learn rather than berate themselves and reinforce a potential negative self-perception.	Keeping it Going visual tools and interactive exercises to explore potential sources of relapse and develop plans to overcome these when they occur.	• Self-monitoring of behaviour • Information about antecedents • Behaviour assessment • Goal setting • Problem solving • Action planning • Review behavioural goals • Restructuring physical and social environment • Avoidance/reducing exposure to cues for behaviour • Reduce negative emotion • Prompts • Remove access to the reward • Framing/reframing • Verbal persuasion about capacity

Reference [19]

### Intervention development

#### Prototype development

Feedback from participants, facilitators, and members of an independent service user group providing a patient and public involvement and engagement contribution throughout the pilot phase was a critical element of the prototype development. A brief summary of the main emergent themes from all three groups of contributors is provided in Tables 3 and 4. Feedback from participants is incorporated in the description of the pilot results below. In the mid-stages of the pilot, when recruitment was critical, service user involvement had a significant input in the development and refinement of the intervention. Concerns raised prior to and during the pilot phase were discussed with service users.

**Table 3:** Feedback from participants and facilitators

Cohort no.	No. of participants	No. of facilitators	Conducted by:	Emergent themes
Cohort 1	*n* = 2 (focus group)	*n* = 3 (Focus group)	1× independent interviewer; 1× scribe	Participants: o Transport a problem—had to catch two buses to get to the venue. Own mental health can get in the way of attending. o Start of the session is ideal—allows time to get to the venue. o Venue convenient to get to, compared to being in town. o All felt comfortable in the group setting. o Liked that they had the chance to learn to lose weight. o Felt happy sharing information. o Some session made them think about what to change. o Booklet difficult to use (both participants dyslexic), especially the sections where they had to write down information. o Some information seemed repetitive. o Learned new information. o Liked how the information was presented on flipcharts and put on the wall. o Incentives (like the pedometer) helped with their confidence to manage their weight. Facilitators: o Concerns about how their time delivering the programme would impact on their other work. o Whole programme felt rushed. o Are sessions too long for participants? o Many queries around organisational support of facilitators.
Cohort 2	–*	*n* = 1 (1:1 interview)	1× independent interviewer	Facilitators: o Felt relaxed and comfortable to deliver. o Curriculum easy to follow. o Still tricky to use the resources. o Noted Next Steps session was too much for one patient in the group—information overload? o Session 2—only one person attend, so activity is difficult to carry out. o Should be a facilitator in reserve in case of an emergency (when facilitator becomes unavailable). o Pleased with the venue. o Storage of resources at the venue potentially an issue. o Incentives a good idea. o Believed intervention should be prescribed like a medication is prescribed. Need for it.
Cohort 3	*n* = 4 (focus group)	*n* = 1 (1:1 interview)	1× independent interviewer; 1 scribe (focus group only)	Participants: o Taxis made it convenient to get to the venue. Three participants emphasised how important this was to them attending and attending on time. o Hesitant at first coming to a group, but like it because people understood them, it was not awkward, no one made them feel bad, it was a small group, and people listened. o Liked consistency of same people delivering. Not having to worry about what people think of them helped them continue to attend. Liked that trained people with delivering. Liked that the group was non-compulsory to attend. o Liked the idea of having a leaflet for people before they sign up to attend, to mention the key good points about the course. o Did not want accompanying people to attend too—wanted to do something for themselves, would make the group that much larger, and would not want to share information with accompanying people. o Information was the right amount—no overload. Wanted more than 4 sessions, up to 10 maybe? o Duration of sessions good—did not want them to be any longer. Liked having a break. Liked lunch, but would have come even if there had not been lunch. o Learned a lot, such as calorie content, benefits of taking more exercise, made you think how to manage yourself, and made you reflect. Enjoyed it. o Liked sessions 3 and 4 best, because of learning about main meals and how to get support outside of the group. o Would have preferred the same two people delivering for the whole course. They become part of the group. Hard to keep opening up to new people every week. o Liked getting ideas from each other. o Enjoyed the incentives. o Changes made: small changes between sessions. Became more aware when shopping. Avoided certain foods. Kept their eye on things—healthier lifestyle. o Wanted more information on how to think and change their attitude. Facilitators: o Harder delivering to larger group. o New facilitator nervous at delivering and not very confident. o Lack of tie for preparation. o Lunch seems to motivate attendance. o Would be good to book transport to avoid excluding people who cannot attend without support. More time to explore participants’ questions. o Training for dealing the questions not covered in the curriculum. o Lack of clarity about responsibility for preparing the room and managing the refreshment arrangements.
Cohort 4	*n* = 5 (focus group)	*n* = 1 (1:1 interview)	1× independent interviewer; 1 scribe (focus group only)	Participants: o Taxis really useful—otherwise would have had to take 1–2 buses o Felt a bit agitated at first because of the size of the group—preferred a group of 6–7 o But did not feel pressured to speak or take part o Duration of course OK but wanted more than 4 weeks. Maybe 6 weeks and a refresher every 6 months o Session length long enough. o Very positive about lunch, meant they did not have to rush to eat before leaving home. o Supporting tools (incentives) very helpful o Liked that there was not much writing in the sessions o Wanted to learn how to manage diet and medication together o Things learned: re-think view of diet and exercise; more about exercise and ways to move forward; frequency, amount, and type, learning to count calories. o Have a leaflet before attending the sessions. Could not remember what they were told by referrers. Include content of sessions and quotes from people who have attended. o No accompanying person, wanted to attend on their own. Also hard to talk about mental illness with accompanying person in the room. Facilitators: o More confident to deliver having done a few sessions already. o Bigger group hard work, but sharing between group members better when group is larger. o Curriculum is useful. o Taxi provision is helping participants attend. o Still some organisation issues with transport. o Not enough time allowed to facilitators to prepare for sessions.
Booster session	*n* = 4 (focus group)	–**	1× independent Interviewer	Participants: o Pleased to be back. o Positive impact of previous sessions—coming back was an encouragement o Good to have prompts and group discussion o Actions made since last session—thinking about what had been learnt when going shopping; looking out for things with less calories and less fat. o Liked Keeping it Going and Action Plan sessions o Duration of booster ‘adequate’ o Would have liked more food samples for food activity o Would have liked pre-made recipe of meals talked about in food activity to take away o Wanted more on exercise in next booster—specifically types of exercise that could be done at home or which did not cost much. Alternatives to going to the gym. o Liked relaxed atmosphere of the session. o Suggested adding in a day out to a park or to a gym, instead of going on their own.

*All three participant did not attend sessions 3 and 4, so no feedback was taken

**This session was delivered by members of the development team

**Table 4:** Feedback from user groups (Leicester)

Groups	No. of users	Conducted by:	Emergent themes
User group 1	*n* = 11	*n* = 4	o Transport: a free bus pass or taxi provided enable/motivate people to attend appointments/sessions, particularly if people are feeling unwell or down. o Duration of a session should not exceed 2 h. Long sessions could cause anxiety, be difficult for people on injections, put them off coming o Sessions should start in the afternoon, around 1:00pm or 2:00pm. o Regular breaks are important for concentration o Would be OK bringing a friend or support worker with them to a session, but not family member. o Barriers to attending sessions: tiredness, depression, feeling isolated or down, bad weather, football match, or something interesting on TV. o Availability of free food/drinks a big motivator. People with schizophrenia do not cook for themselves when they feel down. o Entertainment at the venue is a good motivator, e.g. to play pool. o Call reminders as people forget about appointments. Text or phone OK. o Feedback after the sessions, but a phone call, not a form to fill in. o Many comments on resources, e.g., people are not aware of the amount of sugar in fizzy drinks, which are easy to take especially when people are unwell. o Beer is harder to change—people with schizophrenia can be addicted to alcohol. o Health messages given should depend on the individual
User group 2	1× developer; 1× independent interviewer	2× developers; 1× independent interviewer	o Methods suggested to avoid weight gain: eat food low in calories; have someone to cook for them; carry a bottle of water with them; carry little snacks with them; drink tea at night; use mp3 player to motivate them to walk; have access to recommended information on daily allowances, etc. o People with schizophrenia have no control over craving. o Upsetting events can overwhelm and lead to over eating o Tablets that cause sleepiness can de-motivate to stay physically active. o Do not like the idea of food diaries—a hassle to complete and would probably forget about it. o Exercise seen as good motivator to overcome depression—not about losing weight, but about becoming fitter o Give reward for weight loss, e.g., going away on holiday o It can be hard to take in information and make a change in diet o People did not like the word ‘group’—people with schizophrenia would not attend—replace with ‘drop-in’. o Sessions need to be in a routine, as change is stressful when you have schizophrenia o If people are attending a session for more than one reason, e.g., they are coming to a drop in to get support for weight management, there will be a free lunch and drinks, and there it is possible to play pool and other games at the venue—more are likely to attend.

The aim of the patient and public involvement groups was to gain a better understanding of the logistical and practical issues around attending a programme (e.g., transport) and to collect their thoughts on the layout of sessions (e.g., provision of short comfort breaks). Table 4 lists some of the logistical comments and recommendations, such as sessions should not exceed 2 h; regular breaks are needed (about every 20 min); having a pre-paid taxi service to get to the session is a good motivator; text or phone reminders to attend would help—similar to what is done for doctor’s appointments. This gave a clearer view of how to tailor the intervention to this target group.

The second aim was to have independent feedback on the sessions and resources that had been shortlisted by the team and the co-investigators for use in the intervention (e.g., low calorie drinks activity, recipe book). Service users commented that

- People with schizophrenia know all the information about junk food, but they have ‘no control over craving’
- Change in routine can affect motivation to stay physically active
- Incentives could be given to people who manage to lose weight

These service users also shared their ideas and strategies for eating healthily and staying active, such as using an mp3 player to motivate walking more, carrying a bottle of water when they go out, and taking small snacks with them.

Additionally, contributions from the host mental health team in Sheffield, and from mental health professionals on the study investigator group, resulted in some changes to the prototype curriculum and resources. For example, the development team included appropriate supporting tools (incentives) to participants and reframed some of the language in the curriculum to improve acceptability.

Feedback from the four facilitators involved in the pilot covered some of the same topics, but it was noticeable that a significant proportion of facilitator feedback discussed the support for them to conduct the study and their concerns about delivering the intervention without sufficient support from their organisations, rather than any comments on issues more directly affecting participants.

#### Pilot

##### Recruitment and reminders for attendance

**Fig. 5:**
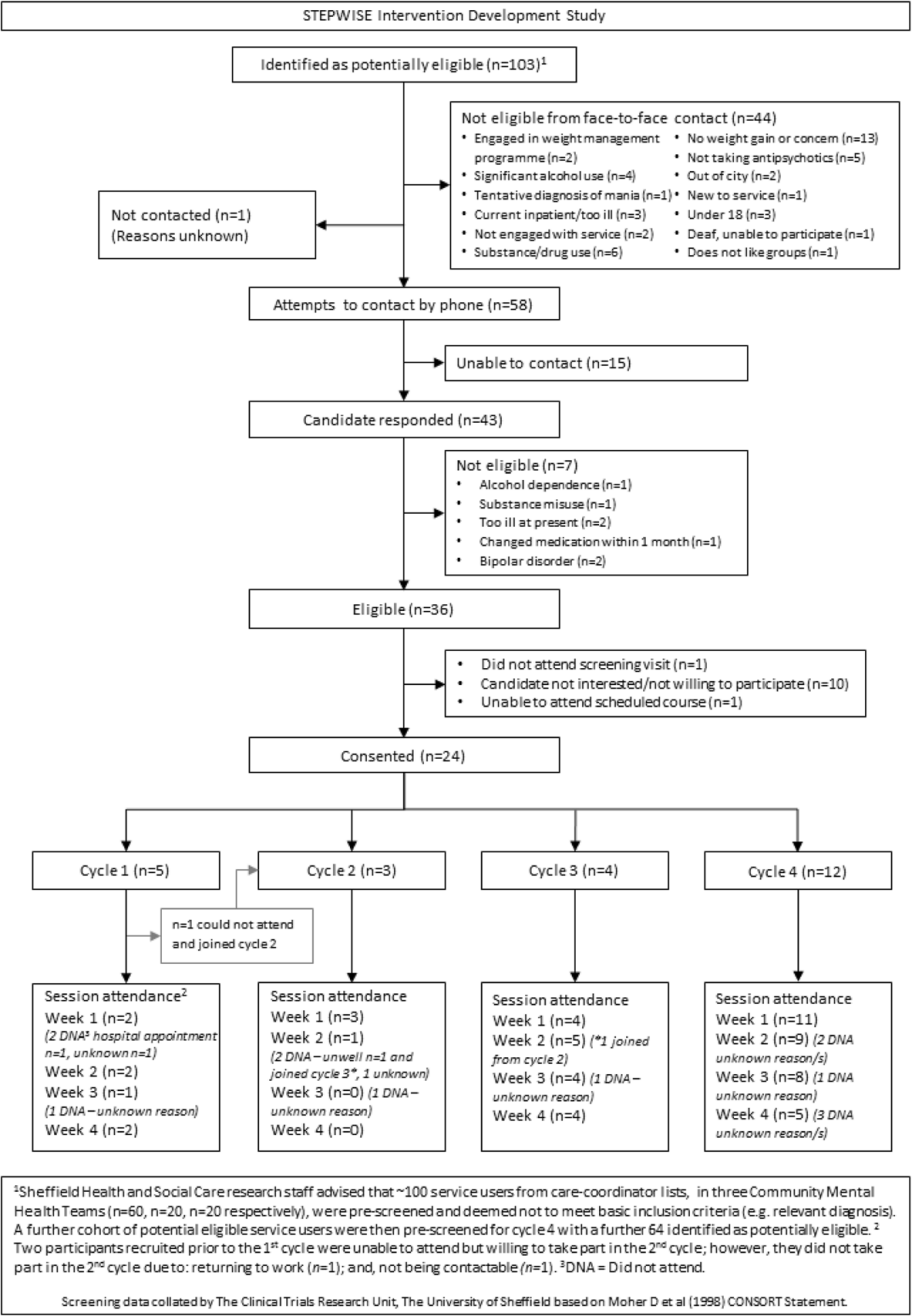
Consort diagram for the STEPWISE pilot

A total of 103 service users with schizophrenia, schizoaffective disorder, or first episode psychosis were approached by the mental health team (Fig. 5). Of these, 44 did not meet the eligibility criteria, 16 were non-contactable and 19 declined. Overall, 24 people consented to take part, of whom 20 attended at least one session.

Recruitment and retention to cohorts 1 and 2 was initially challenging. Following discussion with the intervention development study group, and supported by feedback from the service user group, further recruitment strategies were introduced and basic referral strategies refined to increase attendance in the pilot. This led to greater numbers of participants in cohorts 3 and 4. Whilst people with schizophrenia have similar levels of interest in their physical health to those without psychosis, they struggle to prioritise this over other needs [21]. To recruit successfully, the relevance of the intervention had to be emphasised to potential participants. Text or phone call reminders were introduced to support this, and taxis provided to enable participants to attend sessions.

##### Feedback from participants in the pilot phase

Between January and September 2014, a total of 15 participants gave feedback on their experience of attending pilot sessions at focus groups facilitated by an experienced and independent health psychologist (Table 3). The final two sessions of cohort 2 had no attendees, and so feedback was not taken for that cohort. Feedback was, however, obtained from the booster session pilot. Data derived from these meetings were analysed using principles of the constant comparative approach based on grounded theory [22]. A pragmatic approach was taken to analyse data as it had been obtained only for the purposes of improving the intervention, rather than exploring other themes, such as the effect on participants, which would be part of data gathering in the RCT. The main themes were as follows:

##### Logistics

The practical details around organising the intervention were regarded as important. For example, transportation was noted as fundamental to enabling participants to ‘turn up on time’, and to maintaining high attendance rates. Providing transportation to the venue also reduced the anxiety of travelling for several participants. Problems in arrangements with the taxi service, which had sometimes proved unreliable, were addressed following the second cycle of the pilot. Feedback from cohort 4 included ‘the taxis made me turn up each week’.

The choice of venue was approved as a convenient and familiar place to reach. The time of the sessions (12.30pm for a 1.00pm start) was identified as ideal for participants and the total number of sessions as reasonable in number. Many people with schizophrenia have altered sleep patterns, which can make early morning appointments difficult to attend. The intervention was therefore scheduled at lunchtime and began with provision of a healthy lunch. This meant that sessions were not disrupted if participants arrived late and provided a practical demonstration of how to eat healthily on a limited budget. Participants talked about lunch as ‘nice’ and ‘fabulous’, and for some, it meant not having to rush to eat before coming to the sessions; however, whilst appreciated, some participants said they would have attended the sessions even if lunch had not been provided.

##### Concentration abilities

The literature review had proposed that many people with schizophrenia experience cognitive deficits that could impair their ability to concentrate over prolonged periods of time. In recognition of this finding, and in order to overcome the challenge, participants were made aware that 1–2 breaks would be part of the session, allowance for these were made in the intervention timings and facilitators trained on how to introduce breaks at suitable points in the session, whilst maintaining the momentum of the delivery.

##### Sessions

Overall, feedback demonstrates that the intervention was perceived positively. Table 3 records the main comments. Participants expressed a sense of comfort in being in a small group setting—‘I liked smaller groups’—which enabled them to take in information more efficiently, share information with fellow participants, and also reinforce existing messages. The duration of each session, including breaks where necessary, was ‘adequate’ and ‘just enough’. Participants and facilitators described the use of resources, such as flipcharts, laminates, booklets, and resources as valuable and engaging. Participants reported the benefits of consistency of using the same facilitators throughout the intervention because they became ‘part of the group’, and there was no necessity to keep introducing themselves to new facilitators. Having the same people in the group each time was also a motivator to return: ‘I find it hard to be in a big group in general, but as I got to know the facilitators and others in the group, it helped me to come back’.

##### Incentives and motivation

The introduction of supporting tools, e.g., samples of low calorie drinks and snacks, kitchen and bathroom scales, cookery books, and pedometers, supported the messages provided to participants about the benefits of participation, improved internal motivation, and supported engagement and attendance. The idea of introducing supporting tools was informed by the literature [23].

##### Accompanying person

Contrary to the team’s expectations, there was consensus among participants that they would not benefit from bringing an accompanying person with them to the sessions. Participants felt that taking part in these sessions was something they wanted to accomplish on their own: ‘it needs to be for me and not anyone else”, and that it would be easier to share information with fellow participants with schizophrenia without the presence of people who did not have the condition.

## Discussion

### Key findings

The weight management intervention was positively accepted by facilitators and people with schizophrenia, schizoaffective disorder, and first episode psychosis. Overall, feedback from both these groups was positive, with self-reported behaviour change by the majority of the participants. The importance of a safe and non-judgemental environment in a small familiar setting was strongly highlighted by both participants and facilitators. This development phase has demonstrated that group settings are acceptable, feasible, and have a potential to be effective within this population.

Accessibility is a key issue for people living with severe mental illness. There were concerns that the issues around recruitment experienced in earlier cycles of the pilot study phase would be reflected in the randomised controlled trial. However, the iterative process of feedback from participants and the observation of the facilitators enabled challenges and barriers to be addressed effectively during the pilot, resulting in adaptations which improved recruitment and the researchers’ confidence in uptake and retention in the future RCT.

### Strengths and limitations

During the pilot, a large number of individuals were screened; however, the pilot highlighted challenges regarding the engagement of this population, ranging from collecting biomedical data, assessing eligibility, and ultimately ensuring attendance and ongoing participation in group sessions. The role and suggestions by the patient and public involvement and engagement service user group and the introduction of transportation, lunch, and supporting tools throughout the pilot seemed to have a significant beneficial impact on recruitment and attendance rates, addressing common barriers found in the literature of mental health [23, 24]. The issues raised by participants during the pilot, concurred with findings in the literature highlighting the struggle with weight issues, self-esteem, motivation, and feelings of isolation. This programme dealt with practicalities of weight management, but it also proved beneficial to cover psychological and psychosocial aspects of participants’ wellbeing. Despite evidence to the contrary [25, 26], engagement with this group of participants was achievable and effective.

## Conclusions

This paper describes how a new weight management programme for people with schizophrenia, schizoaffective disorder, and first episode psychosis was developed from first principles. It also describes how the prototype was piloted with service users and with input from potential facilitators (mental healthcare professionals), and how it was subsequently revised in response to the experience of developers, the feedback of participants, service users, and facilitators. The aim of this paper is to highlight the level of detail, the number of iterations, the importance of taking action on feedback from a multiplicity of stakeholders, and the challenges that can be faced when conducting work of this nature, which is infrequently reported in the literature. The intervention was the focus of a recently completed multi-centre randomised controlled trial [27]. We believe that our approach to developing self-management interventions would be applicable to other areas of mental health and in fact any area of health where providing opportunities and supporting individuals to successfully self-manage is appropriate. The key to using our approach successfully is not to seek to replicate the same intervention with alternative content, but to start with the needs of the target participants and what is known from the evidence to achieve an intervention likely to be acceptable and successful.
